# Laboratory Selection Quickly Erases Historical Differentiation

**DOI:** 10.1371/journal.pone.0096227

**Published:** 2014-05-02

**Authors:** Inês Fragata, Pedro Simões, Miguel Lopes-Cunha, Margarida Lima, Bárbara Kellen, Margarida Bárbaro, Josiane Santos, Michael R. Rose, Mauro Santos, Margarida Matos

**Affiliations:** 1 Centro de Biologia Ambiental and Departamento de Biologia Animal, Faculdade de Ciências, Universidade de Lisboa, Lisboa, Portugal; 2 Department of Ecology and Evolutionary Biology, University of California Irvine, Irvine, California, United States of America; 3 Departament de Genètica i de Microbiologia, Universitat Autònoma de Barcelona, Bellaterra, Barcelona, Spain; University of Lausanne, Switzerland

## Abstract

The roles of history, chance and selection have long been debated in evolutionary biology. Though uniform selection is expected to lead to convergent evolution between populations, contrasting histories and chance events might prevent them from attaining the same adaptive state, rendering evolution somewhat unpredictable. The predictability of evolution has been supported by several studies documenting repeatable adaptive radiations and convergence in both nature and laboratory. However, other studies suggest divergence among populations adapting to the same environment. Despite the relevance of this issue, empirical data is lacking for real-time adaptation of sexual populations with deeply divergent histories and ample standing genetic variation across fitness-related traits. Here we analyse the real-time evolutionary dynamics of *Drosophila subobscura* populations, previously differentiated along the European cline, when colonizing a new common environment. By analysing several life-history, physiological and morphological traits, we show that populations quickly converge to the same adaptive state through different evolutionary paths. In contrast with other studies, all analysed traits fully converged regardless of their association with fitness. Selection was able to erase the signature of history in highly differentiated populations after just a short number of generations, leading to consistent patterns of convergent evolution.

## Introduction

The roles of history, chance, and selection in shaping the evolutionary processes of populations adapting to new environments is a long-standing topic of debate in evolutionary biology [1]–[7]. Although repeated adaptive radiations and convergent evolution support the view that evolution is generally predictable [7]–[9], most classic case studies involve morphological traits ([10]–[13], but see [14]). However, the relationships between morphological characters and Darwinian fitness are sometimes ambiguous, suggesting the value of testing for convergence among traits that are more straightforwardly related to fitness. Life-history traits are obvious candidates to study in this respect, because their known association with fitness gives a greater likelihood of interplay between history, chance, and selection that strongly depends on underlying genetic variation [15], [16]. Furthermore, pleiotropy and epistasis are common among life-history traits [17], which should foster the dependence of selection outcome on genetic background [18], [19]. Thus experimental studies of the evolution of life-history characters should allow better tests of whether adaptive convergence occurs when the “tape of evolution” is replayed [20], [21].

Real-time examples of convergent evolution using replicated laboratory populations have been found in several species (e.g. [5], [22]–[28]). But there are also examples of divergence in experimental evolution [29], [30]. However, in all instances where strong convergence was found with selection erasing historical signatures, the lines were recently derived from the same ancestral population and might thus not differ much in their genetic background. Whether historical effects derived from highly and long differentiated ancestral populations do or do not constrain evolution is still an open question (vid. [5]).

A clarification is in order here. The terms convergent evolution and parallel evolution have been used sometimes with the same meaning and sometimes with different meanings (e.g. [31], [32]). A common distinction involves the underlying genetic mechanisms, with the term “convergence” used when the genetic mechanisms involved are different (e.g. in distantly related species) and parallel evolution when the same genetic mechanisms are thought or found to be involved. Here we are interested in phenotypic convergent evolution, and how it is affected by previous history. We will apply the term *convergent* phenotypic evolution to cases where populations start from contrasting adaptive states and evolve such that these differences are erased during adaptation to a common environment. This applies to the real-time evolution studies in bacteria done by Travisano et al. [5] or Melnyk and Kassen [28], or to the reverse evolution studies done by Teotónio et al. [23], [24] among others. By contrast, we refer to examples of *parallel* evolution as those cases which involve populations that are not differentiated to start with or that maintain those differences throughout evolution (e.g. [33]–[35]; see also [36], [37]).

The southern peninsulas of Europe acted as refugia for many species at the height of the last Weichselian ice age (20 kya), species which rapidly expanded northward as the climate warmed. It has become apparent over the last 30 years that many European species are genetically differentiated across at least five major geographic regions as a result of this postglacial expansion [38]. Their life-histories evolved differently due to contrasting environmental conditions, and in some cases differentiated populations have met and produced narrow hybrid zones [38]. This glacial-interglacial climatic reversal provides usefully differentiated wild populations with which to study convergent evolution in replicated laboratory lines derived from such wild populations. We have used the native Palearctic fly *Drosophila subobscura* for this purpose, because it is amenable to laboratory experimentation and exhibits wild genetic differentiation which reflects its postglacial expansion [39]. Moreover, *D. subobscura* exhibits latitudinal clines for body size and chromosomal inversions [40], [41], with recent studies showing that northern populations are becoming more similar to southern populations in response to global warming [42]. Both local adaptation and gene flow may be involved [41], [43].

Our team has been studying the repeatability of adaptive evolution of *D. subobscura* populations to the laboratory environment over repeated samplings from nearby Portuguese locations [26], [44], [45]. For each experimental population, we characterized the evolutionary trajectories of a set of life-history traits - particularly age of first reproduction, early and peak fecundity - as well as a “physiological trait” - starvation resistance (related to fat storage [46]), throughout numerous generations since laboratory introduction. We have shown that, although the laboratory evolution of these populations is repeatable in some respects, evolutionary contingencies often hamper quick convergence across populations [26], [44]. This appears to be due to genetic drift effects during the first generations of laboratory adaptation, since all these populations were derived from the same geographical area, sharing a recent history [45].

However, whether prior history constrains subsequent evolution among geographically differentiated populations is unknown. Taking advantage of the clinal variation of European *D. subobscura* populations, we here report the experimental evolution during the first 22 generations of adaptation to the laboratory of populations derived from wild-caught samples in turn obtained from three contrasting latitudes: Adraga (Portugal), Montpellier (France) and Groningen (Netherlands).

## Materials and Methods

### Founding and Maintenance of populations

*D. subobscura* individuals were collected in August 2010 from three locations in Europe: Adraga (Portugal), Montpellier (France) and Groningen (Netherlands); these were used to start three foundations in the laboratory. The number of founding females was 234 for Adraga (Ad), 171 for Montpellier (Mo) and 160 for Groningen (Gro). F1 eggs and individuals were treated with tetracycline (25 mg/l) and F2 with ceftriaxone and spectinomycin (50 mg/l) due to the presence of pathogenic (not identified) bacteria that caused high larval mortality. No *Wolbachia* was present in founder or control individuals untreated with antibiotics. Females from these first two generations were maintained in separate vials, to equalize their contribution to the next generation. To avoid inbreeding, females were crossed with males from different vials (1^st^ laboratory generation) or derived from a random sample from all vials (2^nd^ generation). At the 3^rd^ generation an equal number of offspring of each female were randomly mixed, giving rise to the outbred populations. At the 4^th^ generation, replicate populations were formed by dividing the overall egg collection of each outbred population in three equal parts (e.g. originating Ad_1_, Ad_2_ and Ad_3_ from the Adraga foundation). Three long established populations (TA – formerly “TW” populations - [26]), derived from a collection in Adraga in 2001, were used as controls and assayed in synchrony with the experimental populations. These populations were in the 115^th^ generation at the time of founding of the newly introduced populations, and were also treated with antibiotics (which led to the new labelling TA) in the same period as the new populations to avoid differences arising from contrasting treatments. In a preliminary assay, no interaction was found between the antibiotic treatment and the different foundations (See Additional Methods S1).

All populations were maintained under the same conditions with synchronous discrete generations of 28 days, reproduction close to peak fecundity, photoperiod of 12 hours of light: 12 hours of darkness at 18°C, with census sizes between 500 and 1200 individuals (with an average census size during the first 22 generations of 782.3 for Ad_1–3_, 607.0 for Mo_1–3_, and 708.6 for Gro_1–3_). Flies were kept in vials with controlled density both for eggs (around 70 eggs per vial) and adults (50 adults per vial). At each generation, flies of a given population emerging from the several vials were thoroughly randomized four to five days after emergence, using CO_2_ anaesthesia. Egg collection for the next generation was done one week later, with flies having between 8 and 12 days of age after emergence (see also [26], [44], [45]).

### Genetic Differentiation

Global genetic differentiation among the three foundations (Adraga, Montpellier and Groningen) was measured at generation 2 using haploid data from chromosomal inversion polymorphisms. Differentiation through *F_ST_* was obtained with Arlequin v3.5 [47].

### Phenotypic assays

Assays were performed at generations 6, 11, 14, 18 and 22 after introduction to the laboratory. Sample sizes per population and assay varied between 15 and 24 mated pairs of flies. Assayed flies were transferred daily and the number of eggs laid per female was counted during the first 12 days. At the 12^th^ day, the flies were transferred to agar medium and starvation resistance was assayed. With this design we estimated three fecundity-related traits: age of first reproduction (number of days between emergence and the first egg laying – ‘A1R’), early fecundity (total number of eggs laid during the first week of life – ‘F1–7’), and peak fecundity (total number of eggs laid between days 8 and 12 – ‘F8–12’). Female starvation resistance was estimated as the number of hours until death (registered every 6 h after transfer to agar – ‘RF’). The latter is a trait strongly related to lipid content, and somewhat correlated with adult survival [46], [48]. Considering the generation time in our populations and the maintenance regime, this trait is not expected to be strongly related to fitness. It may nevertheless have an indirect impact on acquisition and allocation of resources [49]. We also estimated body size (‘BS’) for all assayed females, a morphological trait expected to have effects on life-history traits [50]. As a proxy metric for this trait we used wing size estimated by geometric morphometric analysis (see [51], details in Additional Methods S1).

### Statistical methods

#### Evolutionary trajectories of the several traits

Nested Analyses of Variance (ANOVA) were performed at each generation to test for differences among foundations in all traits. The linear model used was:
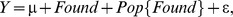
where Y refers to the trait analysed, *Found* refers to the fixed factor Foundations (with three categories, Adraga, Montpellier and Groningen), and *Pop{Found}* refers to the random factor Populations nested in each Foundation (*i.e*, the three replicate populations). Analyses including TA controls were also performed. A type III sum of squares was used in all tests, with the error being the population term. Pairwise comparisons between foundations were performed, with adjustment for multiple testing following the false discovery rate (FDR) procedure of Benjamini and Yekutieli (2001, theorem 1.3 [52]).

The evolutionary trajectory of each trait and foundation was estimated using in each generation the three average values of the replicate populations (as differences from to the average control values); the best linear model being calculated by Type I least-squares linear regression. To test for significance of the overall response across foundations as well as differences in evolutionary rate between them ANCOVA analyses were performed using the linear model:
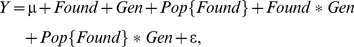
where *Y* refers to the different traits analysed, *Gen* to the generations assayed (as covariate) and the other factors as mentioned above. Analyses using body size as a covariate were also done to account for its effects on other traits.

All data analyses described above were performed using STATISTICA 10 and EXCEL.

#### Multivariate analysis of evolutionary dynamics

A Principal Component Analysis (PCA), using the correlation matrix, was performed with the mean differences between experimental replicate populations and the controls for all studied traits and generations. The multivariate phenotypic trajectories were analysed using the method described in Adams and Collyer [53]. This led to the estimation of differences between pairs of foundations in the following parameters: *magnitude* (differences between the first and the last generations), *direction* (standardized differences between the angles of the first axis of the PCA), and *shape* (deviations of corresponding generations between two scaled and aligned trajectories). To estimate the statistical significance of these differences, 1000 residual-randomization permutations were made. To estimate the distance at generations 6 and 22 between each pair of foundations, we calculated the Euclidean distance using the average scores per foundation for each principal component. In order to calculate the significance of the Euclidean distances, a null distribution was created using 9999 permutations of replicates. Confidence intervals were estimated by 9999 bootstraps at the replicate level within each foundation using the average trait values per replicate. Multivariate analyses were performed using R [54] with the rgl package [55].

#### Estimates of causal components of variation

To analyse the contributions of history, chance, and selection throughout the study, we estimated several variance components for early fecundity and starvation resistance in each generation. We used the nested ANOVA model to estimate the variance components of History – as the differences among foundations - and Chance – as the differences among populations within foundations. To calculate the cumulative effect of Selection for each foundation and generation, we applied a mixed bi-factorial ANOVA. This effect was estimated as the variation between the earliest generation assayed and each one of the later generations. Confidence intervals of variance components were estimated by bootstrap at the level of the error term. For further details, see Additional Methods S1.

## Results

### Early Differentiation

As a measure of the early genetic differentiation between our foundations, we estimated *F_ST_* using chromosomal inversion frequencies. We found highly significant differentiation between foundations at the 2^nd^ generation (*F_ST_* = 0.204, *P*<0.0001).

**Figure 1:**
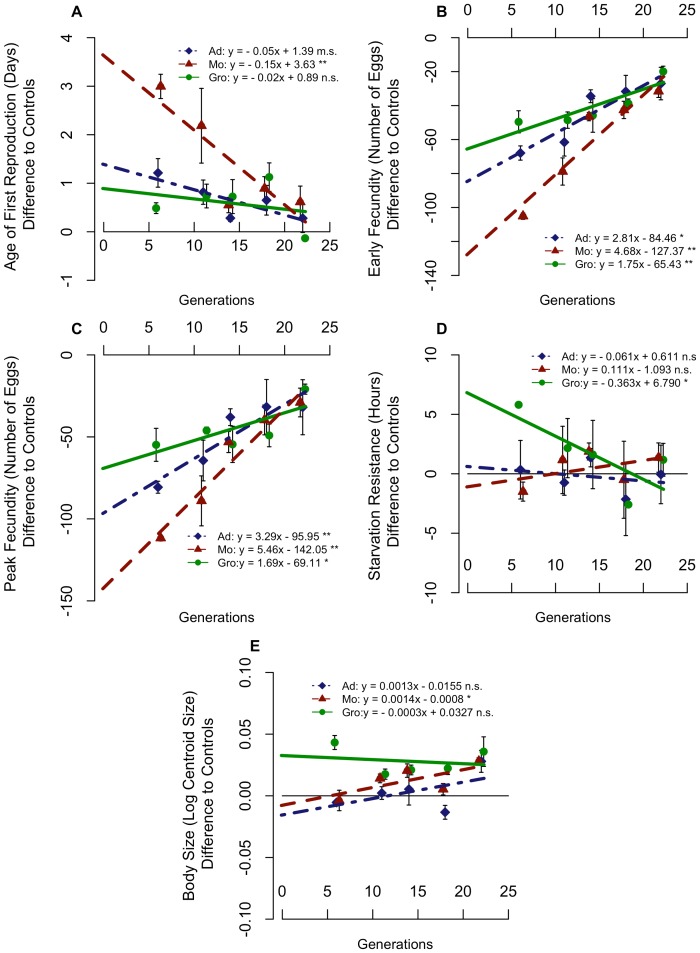
Evolutionary trajectories for the several traits analysed. Average differences from the controls for Age of First Reproduction (A), Early Fecundity (B), Peak Fecundity (C), Starvation Resistance (D) and Body Size (E) are presented for each foundation, as well as the corresponding linear regression models. Error bars correspond to variation between replicate populations of each foundation. Significance levels: *P*>0.1 n.s.; 0.1>*P*>0.05 m.s.; 0.05>*P*>0.01*; 0.01>*P*>0.001**; P<0.001***.

All foundations were clearly differentiated in phenotypic traits at the 6^th^ generation, with Groningen females having better performance for all life-history traits as well as female starvation resistance (Fig. 1, Table S1). Additionally, Groningen females had a significantly higher body size compared to other foundations.

### Evolutionary trajectories of single traits

The initially low fecundity (and high age of first reproduction) of the three foundations quickly improved and they phenotypically converged through time, such that they were no longer significantly different by the 14^th^ generation (Fig. 1A–C, Table S1a and b). By the 22^nd^ generation, fecundity traits did not differ significantly between the foundations and the control baseline, with the exception of Montpellier-derived populations for early fecundity (Fig. 1B, Table S1c). By contrast, starvation resistance was initially about equal or higher (Groningen) than the control baseline, and quick convergence was observed because of a drop in starvation resistance among Groningen-derived flies (Fig. 1D). In fact, by the 11^th^ generation the foundations were no longer differentiated between them or relative to the controls (Table S1). Convergence of the three foundations by means of different evolutionary rates can also be seen from the significant foundation*generation interaction term in the global ANCOVA and pairwise tests (see Table S2).

Convergence in body size was also observed among the three foundations, with the relative values of Montpellier and Adraga (measured as differences from the controls) increasing towards the values of Groningen, which was stable through time (Fig. 1E). This is seen in the contrasting smaller sizes of Adraga and Montpellier populations at the 6^th^ generation, while by the 22^nd^ generation all foundations had very similar sizes. Nevertheless, Adraga did not show a significant linear trend, or differences in evolutionary rate relative to Groningen, and differences between Montpellier and Groningen were only marginally significant (Fig 1E, Table S2b). Interestingly, foundations did not converge in body size to the control values (Fig. 1E, Table S1c). In absolute terms both Groningen and the controls decreased in body size with time (from G6 to G22 Groningen declined 2.9% and TA declined 2.4%), while both Adraga and Montpellier populations remained fairly stable (increases of 0.40% and 0.27%, respectively). We are assuming, as is common in experimental evolutionary designs that the long-established, TA populations, are close to stable genetic equilibrium, serving as controls [26], [56]. Thus the temporal changes presented by these populations are likely environmental in origin and common to all foundations. Therefore differences from TA populations will give the evolutionary patterns of our experimental populations. Size differences did not account for the various evolutionary patterns in either fecundity or starvation resistance, as analyses defining body size as covariate led to the same conclusions (Tables S2 and S3).

Consistently for all traits, we found that the populations showing a larger early differentiation also exhibited a higher evolutionary rate (Fig. 1).

### Multivariate evolutionary trajectories

**Figure 2:**
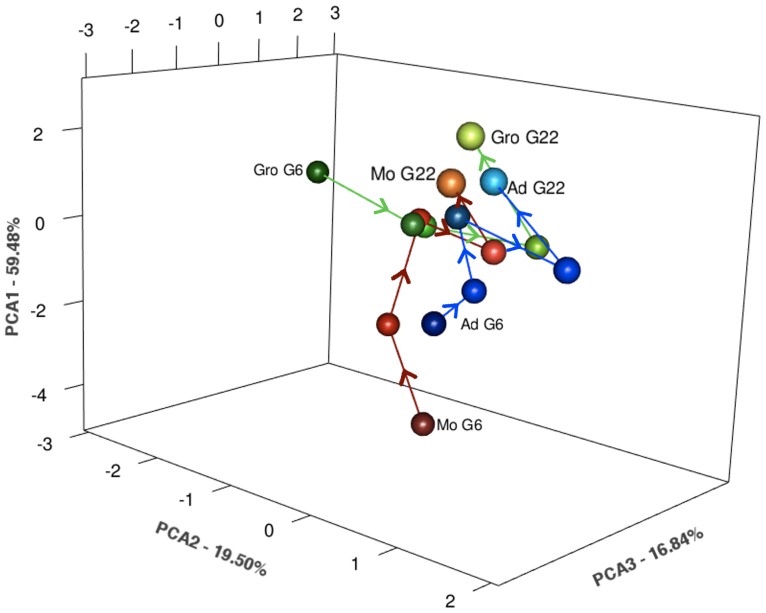
Multivariate evolutionary trajectories using Principal Component Analysis. All traits, generations and foundations were included.

We integrated all phenotypic traits using Principal Component Analysis (PCA) to plot the multivariate evolutionary trajectories of the several populations (see Fig. 2 and Methods). The first axis of the PCA refers to changes involving life-history traits. The second and third axes show changes in starvation resistance and body size, respectively, possibly as correlated responses to selection (Table S4a).

We also estimated three parameters to compare the multivariate trajectories across populations: magnitude (rate of response), direction (convergence *vs* divergence), and shape (evolutionary path). Populations showed significant differences in magnitude and shape but not in direction, suggesting a clear convergence to the same adaptive state, though with contrasting routes and rates (Fig. 2, Table 1 and S4). Convergence was also confirmed by estimating the Euclidean distances between populations, which gave significant values in the initial generation, while by generation 22 they were no longer significantly higher than would be expected by chance alone (Fig. 2, Table S4b). Euclidean distance between all foundations decreased significantly between generation 6 and generation 22 (Table S4b).

**Table 1:** Pairwise differences and significance levels using Multivariate trajectory analysis.

Parameter	Found	Ad	Gro
Magnitude	Ad	—	—
	Gro	16.235 n.s.	—
	Mo	74.245 m.s.	90.480 *
Orientation	Ad	—	—
	Gro	2.532 n.s.	—
	Mo	1.708 n.s.	3.818 n.s.
Shape	Ad	—	—
	Gro	0.739 **	—
	Mo	0.178 n.s.	0.716 **

Note: significance levels: *P*>0.1 n.s.; 0.1>*P*>0.05 m.s.; 0.05>*P*>0.01*; 0.01>*P*>0.001**.

Magnitude refers to the amount of evolutionary response, Orientation to the direction of the evolutionary path and Shape to the route of this path.

### The effect of History, Chance and Selection

**Figure 3:**
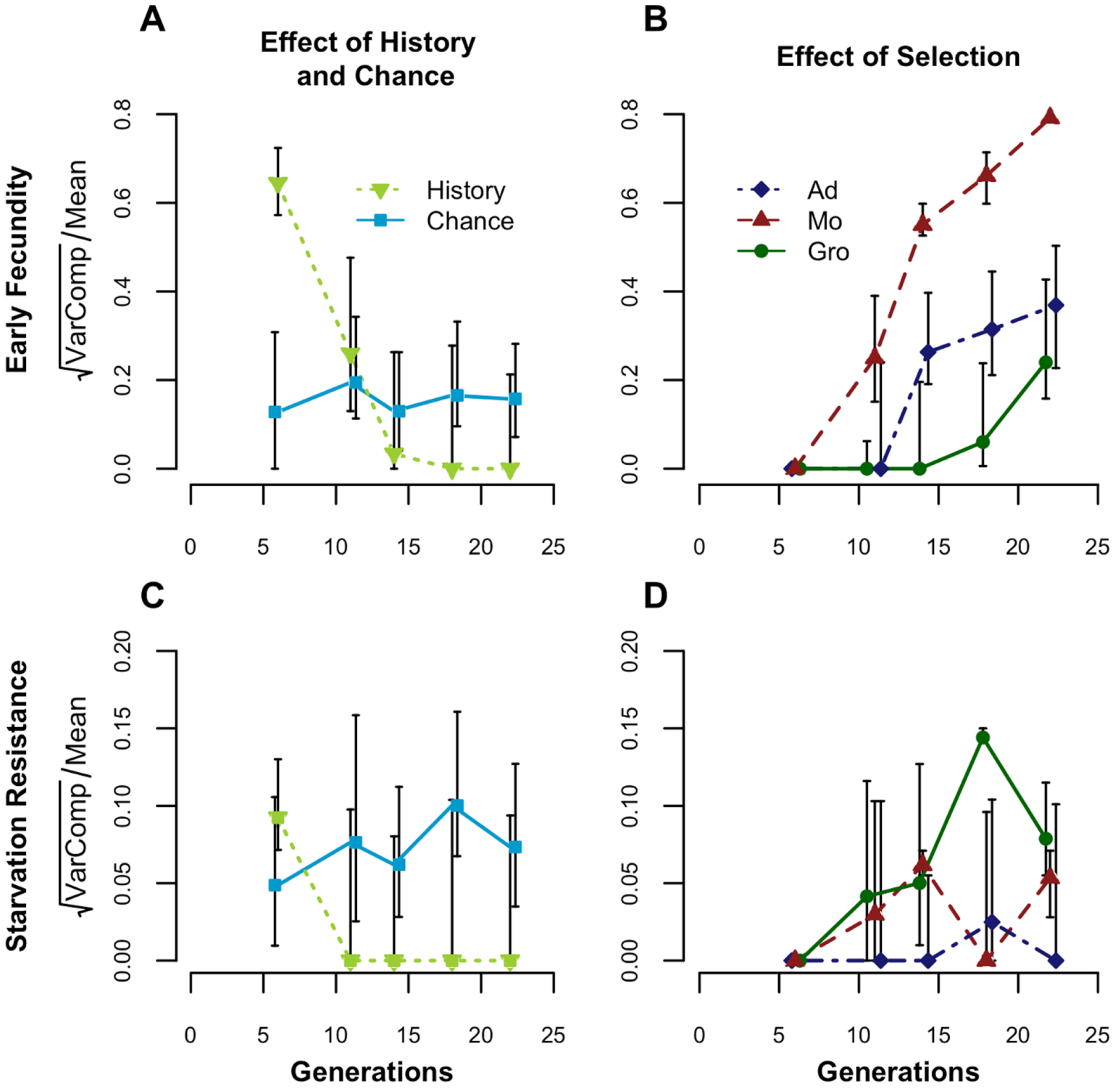
Variance components through time for history, chance and selection. Values presented are for Early Fecundity (A, B) and Starvation Resistance (C, D). Bars represent 95% confidence limits (see Material and Methods, Additional Methods S1 and Tables S5 and S6 for details).

Knowing that these populations converged to the same phenotypic state using different routes and rates, we measured the effects of history, chance and selection through time (Fig. 3). We focused this analysis on early fecundity and starvation resistance because of their contrasting associations with fitness (see Fig. 1B and 1D and [26], [44]). Strong historical differentiation among foundations for early fecundity quickly faded as selection produced convergence after only 14 generations (Fig. 3, Tables S5 and S6). The initial historical variation for early fecundity was considerably higher than the variation due to sampling effects alone that was found for previous foundations from nearby locations. In fact, variance components estimated from our previous studies in 2001 and 2005 as a measure of sampling effects [26], [45] were at least eight times lower than the variance estimated among foundations in the present study (see Table S7).

A similar evolutionary pattern was observed for starvation resistance, starting from less differentiation (Fig. 3). However, when comparing the effect of chance to historical differentiation, there was a clear contrast between traits, particularly at generation 6 (ratio of history/chance  = 25.3 for early fecundity and 3.6 for starvation resistance). Furthermore, whereas for early fecundity the role of chance was relatively small, for starvation resistance chance had similar effects to those of initial history. This suggests a bigger role for chance events during the evolution of starvation resistance (see Fig. 3A and 3C, Table S5).

The contrasting rates of convergence among foundations can be seen in the temporal changes of cumulative selective response for early fecundity (Fig. 3B). Higher temporal heterogeneity was observed for starvation resistance, with only the Groningen-derived populations changing markedly through time (see Table S6, Fig. 3D).

## Discussion

In order to disentangle the relative impact of historical factors and selection during adaptation, we performed a real-time evolution study of highly differentiated *Drosophila subobscura* populations in nature during adaptation to a new environment under controlled conditions. Here we directly quantified the relative contribution of history and selection, an approach that has been used mostly in asexual organisms ([5], [28], , but see [25]). Combining this approach and the analysis of evolutionary multivariate trajectories we found a clear pattern of convergence despite the high level of differentiation in the initial foundations, suggesting that historical contingencies did not play a major role in adaptation.

Previous studies of experimental evolution, both in sexual and asexual populations, have also found convergence patterns, although these patterns were less pervasive across traits [5], [22], [24]–[26], [44]. Our results contrast with several studies in asexual populations, where history played an important role in traits weakly related to fitness [5], [57] or even in traits strongly selected for [58]. The incomplete convergence observed by Spor et al [58] in *Saccharomyces cerevisiae* (initially highly differentiated) may have been due to the fact that their populations were still adapting at the end of the study. In our present study, the strong signature of history was quickly erased across all traits regardless of their association with fitness. It is possible that history has a greater impact in asexual populations, perhaps due to their relative lack of standing genetic variation and negative epistasis [23]. Blount et al [59] showed that historical contingencies have an important effect during adaptation in *Escherichia coli*, allowing the species to explore new ecological opportunities (see also [60] for an example in viruses). Furthermore, chance genetic associations during the initial stage of adaptation may even lead to divergence between asexual populations in traits not relevant to fitness [61]. Some experimental studies in sexual populations have also shown historical contingencies that prevented convergence (e.g. [29], [30], [62]–[65]), which illustrates the dangers of generalization.

We have previously shown that stochastic events during the early stages of laboratory colonization have some impact in the adaptive dynamics of *D. subobscura* populations sampled from nature in near-by or even the same location [26], [44], [45]. This might suggest an overall role of evolutionary contingencies hampering convergence. Nevertheless, the present study shows that strong initial differentiation among populations does not prevent convergent evolution. Our results differ from those abovementioned studies where populations of *Drosophila* derived from contrasting latitudes showed parallel [65] or even divergent [29], [30] evolution under uniform selection. While the lack of convergence in those studies might be due to multiple solutions to the same problem [18], it is possible that the use of a limited number of isofemale lines in those studies contributed to their divergent outcomes.

The clear pattern of convergence across all traits in our study also suggests that the populations evolved to the same adaptive equilibrium. It is an open question whether this observed pattern of convergent adaptation might not have occurred if we had used a different laboratory environment. In fact, Melnyk and Kassen [28] showed contrasting evolutionary patterns in the same *Pseudomonas fluorescens* populations adapting to two different laboratory environments. These patterns point to the possibility of different underlying adaptive landscapes and different contributions of history *vs* selection, among selection regimes. Whether this applies to sexual populations with contrasting histories across fitness-related traits and ample standing genetic variation such as ours remains to be seen. Additionally, it is worth noting that our populations evolved in a benign homogeneous environment, and it is an open question whether other evolutionary outcomes may emerge as a function of the complexity and harshness of the environment [33], [66].

In a study of reverse evolution in *Drosophila melanogaster*, Teotónio et al. [22], [24] found that convergence to the ancestral state was not universal across traits, but instead depended somewhat on history as a result of previous adaptation to different selective regimes. It may be the case that adaptation to a novel environment, such as ours, is fostered by positive additive variance/covariance matrices across traits [49], [67]. In the case of Teotónio et al's. study, the hypothesis that genetic variation affecting adaptation to the lab environment may have been exhausted is not likely, given the genome-wide absence of selective sweeps leading to low heterozygosity found by Burke et al. [68] in their study of ten of those populations. Antagonistic pleiotropy seems to have played a role in our Groningen populations, since starvation resistance quickly declined during adaptation of those populations. This possible trade-off between starvation resistance and other traits, found only in Groningen, suggests that different mechanisms/associations among traits may be involved, in spite of the general convergence across foundations. Differential mechanisms of acquisition versus allocation of resources might have had different impacts between foundations or can change during the different phases of adaptation [56]. In this regard it is tempting to suggest that the Groningen populations were already better suited to the laboratory environment. Alternatively relaxed selection might have also contributed, though it is unlikely that it could by itself lead to the quick evolutionary pattern observed. Future studies on the effect of specific environments on G matrices would be interesting.

The fact that we report here full phenotypic convergence in only 22 generations of adaptation calls for a word of caution, in the sense that these populations might diverge if they continue to evolve at a steady rate in the future. This is not a likely scenario, considering the evidence for a slowing down of evolutionary rates of adaptation in our longer-term studies of laboratory adaptation in populations like these (e.g. [69], [70]). Of course, close study of these populations after more generations of laboratory adaptation would settle this issue. More generations may also help to clarify the evolutionary dynamics of body size. Though convergence occurred among foundations, they did not evolve towards the values of the long established, control populations. This may be due to complex trade-offs that might manifest at a later evolutionary phase [56]. Alternatively, founder events or genetic drift may maintain these differences.

It is important to note that convergence at the phenotypic level does not necessarily imply that the underlying genetic basis of convergence is uniform. Although some studies show convergent (or parallel) genotypic evolution [9], [36], [71], [72], phenotypically convergent (or parallel) evolution through different genetic mechanisms has also been found in both sexual and asexual organisms [27], [31], [32], [73]–[75]. A plethora of factors could be responsible for these varying genetic pathways: different levels of standing genetic variation, mutational input, epistasis and pleiotropic effects [23], [27], [32], [73], [74], [76]. It will be interesting to analyse to what extent the fast phenotypic convergence of our populations is matched at the genomic level.

The results of our study highlight the potential for error in characterizing geographical patterns from comparisons of populations even after relatively few generations of laboratory adaptation. Here we show that starvation resistance started with higher values for the Groningen foundations but quickly converged to values similar to those of the other populations. The absence of a latitudinal European cline for this trait in the study by Gilchrist et al. [77] might thus be due to laboratory convergent evolution during the multiple generations of laboratory culture prior to their measurement of starvation resistance.

To sum up, we found that populations with clear initial differentiation quickly converged phenotypically during adaptation to a new, common laboratory environment. Thus, selection was able to quickly overcome the effects of history even in laboratory populations founded from populations highly differentiated in nature. In this sense, phenotypic evolution was generally predictable, even across a set of complex traits, and was not significantly dependent on chance events or historical constraints.

## Supporting Information

Table S1 **Analyses of differences in life-history traits at each generation among and within foundations.**
(DOCX)

Table S2 **ANCOVA models for each trait with Generation as covariate.**
(DOCX)

Table S3 **ANCOVA models for each trait with Generation and Body Size as covariates.**
(DOCX)

Table S4 **Principal Component Analysis including all traits and generations.**
(DOCX)

Table S5 **Variance components of History and Chance for Early Fecundity and Starvation Resistance.**
(DOCX)

Table S6 **Variance components of Selection for Early Fecundity and Starvation Resistance.**
(DOCX)

Table S7 **Comparisons of the initial differences between the three foundations, relative to differences in previous studies.**
(DOCX)

Additional Methods S1 (DOCX)
