# Supplementary material for: Laboratory Selection Quickly Erases Historical Differentiation
Source: PLoS One. 2014 May 2;9(5):e96227. doi: 10.1371/journal.pone.0096227 (PMC4008540; doi:10.1371/journal.pone.0096227)
Supplement: Table S1 — Analyses of differences in life-history traits at each generation among and within foundations. (DOCX) [file pone.0096227.s001.docx]

**Table S1.** Analyses of differences in life-history traits at each generation among and within foundations (Adraga; Montpellier and Groningen). The Global ANOVA model (A) and the paired comparisons between foundations (B) and between foundations and TA controls (C) are presented.

A)

|  |  | Age of First Reproduction | | Early Fecundity | | Peak Fecundity | | Starvation Resistance | | Body Size | |
| --- | --- | --- | --- | --- | --- | --- | --- | --- | --- | --- | --- |
| Gen | Model parameters | MS | F_(df1, df2)_ | MS | F_(df1, df2)_ | MS | F_(df1, df2)_ | MS | F_(df1, df2)_ | MS | F_(df1, df2)_ |
| 6 | Found | 117.795 | F_2,6_ = 30.773 *** | 57088.969 | F_2,6_ = 37.886 *** | 55601.898 | F_2,6_ = 19.889 ** | 978.532 | F_2,6_ = 6.664 * | 0.052 | F_2,6_ = 23.869 *** |
|  | Pop(Found) | 3.827 | F_6,203_= 0.790 n.s | 1507.032 | F_6,203_ = 1.942 m.s. | 2798.664 | F_6,195_ = 2.336 * | 147.015 | F_6,190_ = 2.096 m.s. | 0.002 | F_6,187_ = 1.750 n.s. |
|  | Error | 4.842 |  | 776.054 |  | 1198.087 |  | 70.133 |  | 0.001 |  |
| 11 | Found | 35.264 | F_2,6_ = 2.766 n.s. | 12215.487 | F_2,6_ = 4.559 m.s. | 22354.86 | F_2,6_ = 3.668 m.s. | 93.415 | F_2,6_ = 0.388 n.s. | 0.003 | F_2,6_ = 3.418 n.s. |
|  | Pop(Found) | 12.752 | F_6,148_ = 2.703 * | 2680.802 | F_6,147_ = 2.980 ** | 6115.223 | F_6,128_ = 3.529 ** | 241.342 | F_6,128_ = 2.276 * | 0.001 | F_6,130_ = 0.690 n.s. |
|  | Error | 4.717 |  | 899.527 |  | 1732.707 |  | 106.055 |  | 0.001 |  |
| 14 | Found | 2.622 | F_2,6_ = 1.029 n.s. | 2403.769 | F_2,6_ =1.136 n.s. | 4380.752 | F_2,6_ = 1.365 n.s. | 3.553 | F_2,6_ = 0.023 n.s. | 0.004 | F_2,6_ = 1.107 n.s. |
|  | Pop(Found) | 2.55 | F_6,148_ = 2.171 * | 2115.843 | F_6,150_ = 2.428 * | 3164.982 | F_6,145_ = 2.196 * | 154.364 | F_6,145_ = 3.349 ** | 0.004 | F_6,141_ = 4.860 *** |
|  | Error | 1.175 |  | 871.484 |  | 1441.296 |  | 46.092 |  | 0.001 |  |
| 18 | Found | 2.755 | F_2,6_ = 0.658 n.s. | 1577.957 | F_2,6_ = 0.522 n.s. | 3640.604 | F_2,6_ = 0.551 n.s. | 61.055 | F_2,6_ = 0.187 n.s. | 0.015 | F_2,6_ = 12.204 ** |
|  | Pop(Found) | 4.187 | F_6,143_ = 1.065 n.s. | 2180.378 | F_6,145_ = 2.268 * | 6634.753 | F_6,141_ = 3.277 ** | 327.784 | F_6,141_ = 4.587 *** | 0.001 | F_6,138_ = 1.384 n.s. |
|  | Error | 3.931 |  | 961.267 |  | 2024.798 |  | 71.456 |  | 0.001 |  |
| 22 | Found | 9.59 | F_2,6_ = 2.081 n.s. | 2344.447 | F_2,6_ =0.880 n.s. | 2177.33 | F_2,6_ = 0.254 n.s. | 39.825 | F_2,6_ = 0.184 n.s. | 0.001 | F_2,6_ = 0.274 n.s. |
|  | Pop(Found) | 4.612 | F_6,195_ = 1.784 n.s. | 2666.833 | F_6,196_ = 2.974 ** | 8585.468 | F_6,194_ = 3.926 *** | 216.429 | F_6,194_ = 3.981 *** | 0.005 | F_6,191_ = 6.784 *** |
|  | Error | 2.585 |  | 896.703 |  | 2186.884 |  | 54.359 |  | 0.001 |  |

Note: significance levels: *P*>0.1 n.s.; 0.1>*P*>0.05 m.s.; 0.05>*P*>0.01*; 0.01>*P*>0.001**; *P*<0.001 ***

B)

| Gen | Paired Comparisons | Age of First Reproduction | Early Fecundity | Peak Fecundity | Starvation Resistance | Body Size |
| --- | --- | --- | --- | --- | --- | --- |
| 6 | Ad vs Mo | 29.043 ** | 32.065 ** | 11.313 * | 0.736 | 0.036 |
|  | Ad vs Gro | 4.901 m.s, n.s. | 7.892 *, m.s. | 8.218 *, m.s. | 6.884 *, m.s. | 36.318 ***,†† |
|  | Mo vs Gro | 58.379 *** | 73.152 *** | 39.708 ***, †† | 12.0784 * | 33.266 ** |
| 11 | Ad vs Mo | 3.856 m.s, n.s. | 2.878 | 2.380 | 0.338 | 3.802 m.s, n.s. |
|  | Ad vs Gro | 0.013 | 1.608 | 1.082 | 0.756 | 6.300 *, m.s. |
|  | Mo vs Gro | 4.359 m.s, n.s. | 9.040 * | 7.202 *, m.s. | 0.098 | 0.334 |
| 14 | Ad vs Mo | 0.745 | 1.786 | 1.877 | 0.046 | 1.588 |
|  | Ad vs Gro | 2.023 | 1.618 | 2.228 | 0.012 | 1.677 |
|  | Mo vs Gro | 0.311 | 0.006 | 0.017 | 0.011 | 0.002 |
| 18 | Ad vs Mo | 0.365 | 1.413 | 0.246 | 0.209 | 7.065 *, m.s. |
|  | Ad vs Gro | 1.315 | 0.575 | 1.096 | 0.014 | 24.298 ** |
|  | Mo vs Gro | 0.329 | 0.166 | 0.333 | 0.328 | 5.594 m.s, n.s. |
| 22 | Ad vs Mo | 0.824 | 0.263 | 0.033 | 0.314 | 0.000 |
|  | Ad vs Gro | 1.243 | 0.640 | 0.471 | 0.233 | 0.401 |
|  | Mo vs Gro | 4.146 m.s, n.s. | 1.732 | 0.261 | 0.005 | 0.418 |

Note: F values with 1 degree of freedom for Foundation and 6 for the Error term (Population) are presented. Significance levels: 0.1>*P*>0.05 m.s; 0.05>*P*>0.01*; 0.01>*P*>0.001**; *P*<0.001 ***. Significance levels were also corrected for multiple comparisons using the False Discovery Rate adjustment (see Material and Methods), and are presented whenever changes in significance relative to uncorrected values occur: 0.055>*P*>0.027 m.s. (for 0.1>α>0.05); 0.027>*P*>0.005† (for 0.05>α>0.01); 0.005>*P*>0.0005†† (for 0.01>α>0.001). For non-significant F values (*P*>0.055 n.s.) no indication of significance is given (except when test becomes non significant after FDR adjustment).

C)

| Gen | Paired Comparisons | Age of First Reproduction | Early Fecundity | Peak Fecundity | Starvation Resistance | Body Size |
| --- | --- | --- | --- | --- | --- | --- |
| 6 | Ad vs TA | 18.128 ** | 127.866 *** | 72.336 *** | 0.031 | 0.496 |
|  | Mo vs TA | 110.505 *** | 310.505 *** | 138.431 *** | 0.589 | 0.239 |
|  | Gro vs TA | 2.953 | 69.520 *** | 34.940 *** | 9.413 * | 34.222 *** |
| 11 | Ad vs TA | 1.82 | 43.462 *** | 16.537 ** | 0.057 | 0.093 |
|  | Mo vs TA | 13.435 **,† | 74.070 *** | 35.848 *** | 0.155 | 3.906 m.s., n.s. |
|  | Gro vs TA | 1.496 | 27.580 ***, †† | 9.234 * | 0.513 | 6.016 *, m.s. |
| 14 | Ad vs TA | 1.025 | 11.338 **, † | 8.220 * | 0.234 | 0.262 |
|  | Mo vs TA | 3.890 m.s., n.s. | 20.411 ** | 15.764 ** | 0.441 | 3.648 m.s. n.s. |
|  | Gro vs TA | 6.745 *, m.s. | 20.199 ** | 16.457 ** | 0.325 | 3.788 m.s., n.s. |
| 18 | Ad vs TA | 3.211 | 8.508 * | 2.993 | 0.426 | 2.145 |
|  | Mo vs TA | 6.353 *, m.s. | 15.842 ** | 4.864 m.s, n.s. | 0.024 | 0.347 |
|  | Gro vs TA | 9.479 * | 12.504 **, † | 6.981 *, m.s. | 0.603 | 5.741 *, m.s. |
| 22 | Ad vs TA | 0.571 | 7.095 *, m.s. | 4.400 m.s., n.s. | 0.001 | 6.095 *, m.s. |
|  | Mo vs TA | 2.808 | 9.757 * | 3.704 m.s., n.s. | 0.352 | 6.492 *, m.s. |
|  | Gro vs TA | 0.132 | 3.857 m.s., n.s. | 1.886 | 0.257 | 10.368 * |

Note: See footnote above (Table S1b) for details on significance levels and multiple test corrections (see also Material and Methods).
