# Supplementary material for: Laboratory Selection Quickly Erases Historical Differentiation
Source: PLoS One. 2014 May 2;9(5):e96227. doi: 10.1371/journal.pone.0096227 (PMC4008540; doi:10.1371/journal.pone.0096227)
Supplement: Table S2 — ANCOVA models for each trait with Generation as covariate. (DOCX) [file pone.0096227.s002.docx]

**Table S2**. ANCOVA models for each trait with Generation as covariate.

A) Global ANCOVA model

| Trait | Model parameters | MS | F |
| --- | --- | --- | --- |
| Age of First Reproduction | Gen | 7.9 | F_1,6_ = 42.266 *** |
|  | Found*Gen | 2.2 | F_2,6_ = 11.804 ** |
|  | Pop(Found)*Gen | 0.2 | F_6,27_ = 0.394 n.s. |
|  | Error | 0.5 |  |
| Early Fecundity | Gen | 13071.1 | F_1,6_ = 88.998 *** |
|  | Found*Gen | 1007.6 | F_2,6_ = 6.861 * |
|  | Pop(Found)*Gen | 146.9 | F_6,27_ = 1.016 n.s. |
|  | Error | 144.6 |  |
| Peak Fecundity | Gen | 16675.7 | F_1,6_ = 42.171 *** |
|  | Found*Gen | 1640.3 | F_2,6_ = 4.148 m.s. |
|  | Pop(Found)*Gen | 395.4 | F_6,27_ = 1.338 n.s. |
|  | Error | 295.4 |  |
| Starvation Resistance | Gen | 15.0 | F_1,6_ = 4.970 m.s. |
|  | Found*Gen | 26.4 | F_2,6_ = 8.765 * |
|  | Pop(Found)*Gen | 3.0 | F_6,27_ = 0.295 n.s. |
|  | Error | 10.2 |  |
| Body Size | Gen | 0.0009 | F_1,6_ = 4.875 m.s. |
|  | Found*Gen | 0.0005 | F_2,6_ = 2.421 n.s. |
|  | Pop(Found)*Gen | 0.0002 | F_6,27_ = 0.866 n.s. |
|  | Error | 0.0002 |  |

Note: significance levels: *P*>0.1 n.s.; 0.1>*P*>0.05 m.s.; 0.05>*P*>0.01*; 0.01>*P*>0.001**; *P*<0.001 ***

B) Paired comparisons between foundations

| Trait | Comparison | F_1,6_ |
| --- | --- | --- |
| Age of First Reproduction | Ad vs Mo | 12.680 * |
|  | Ad vs Gro | 1.172 n.s. |
|  | Mo vs Gro | 21.560 ** |
| Early Fecundity | Ad vs Mo | 5.456 m.s., n.s. |
|  | Ad vs Gro | 1.747 n.s. |
|  | Mo vs Gro | 13.379 * |
| Peak fecundity | Ad vs Mo | 2.720 n.s. |
|  | Ad vs Gro | 1.490 n.s. |
|  | Mo vs Gro | 8.235 *, m.s. |
| Starvation Resistance | Ad vs Mo | 2.234 n.s. |
|  | Ad vs Gro | 6.967 *, m.s. |
|  | Mo vs Gro | 17.092 **, † |
| Body Size | Ad vs Mo | 0.016 n.s. |
|  | Ad vs Gro | 3.386 n.s. |
|  | Mo vs Gro | 3.861 m.s., n.s. |

Note: F values with 1 degree of freedom for Foundation*Generation and 6 for the Error term (Population(Foundation)*Generation) are presented. Significance levels: *P*>0.1 n.s.; 0.1>*P*>0.05 m.s.; 0.05>*P*>0.01*; 0.01>*P*>0.001**. Significance levels were also corrected for multiple comparisons using False Discovery Rate adjustment (see Material and Methods), and are presented whenever changes in significance relative to uncorrected values occur: *P*>0.055 n.s.; 0.055>*P*>0.027 m.s. (for 0.1>α>0.05); 0.027>*P*>0.005† (for 0.05>α>0.01).
