# Supplementary material for: Laboratory Selection Quickly Erases Historical Differentiation
Source: PLoS One. 2014 May 2;9(5):e96227. doi: 10.1371/journal.pone.0096227 (PMC4008540; doi:10.1371/journal.pone.0096227)
Supplement: Table S3 — ANCOVA models for each trait with Generation and Body Size as covariates. (DOCX) [file pone.0096227.s003.docx]

**Table S3.** ANCOVA models for each trait with Generation and Body Size as covariates.

| Trait | Model parameters | MS | F |
| --- | --- | --- | --- |
| Age of First Reproduction | Gen | 5.2 | F_1,6_ = 21.613 ** |
|  | Found*Gen | 1.6 | F_2,6_ = 7.359 * |
|  | Pop(Found)*Gen | 0.2 | F_6,26_ = 0.460 n.s. |
|  | Body Size | 0.9 | F_1,26_ = 1.877 n.s. |
|  | Error | 0.5 |  |
| Early Fecundity | Gen | 10921.6 | F_1,6_ = 72.882 *** |
|  | Found*Gen | 859.1 | F_2,6_ = 5.731 * |
|  | Pop(Found)*Gen | 149.9 | F_6,26_ = 1.005 n.s. |
|  | Body Size | 24.3 | F_1,26_ = 0.163 n.s. |
|  | Error | 149.3 |  |
| Peak Fecundity | Gen | 15094.1 | F_1,6_ = 41.161 *** |
|  | Found*Gen | 1597.9 | F_2,6_ = 4.302 m.s. |
|  | Pop(Found)*Gen | 374.3 | F_6,26_ = 1.222 n.s. |
|  | Body Size | 48.7 | F_1,26_ = 0.159 n.s. |
|  | Error | 306.3 |  |
| Starvation Resistance | Gen | 25.2 | F_1,6_ = 6.612 * |
|  | Found*Gen | 15.0 | F_2,6_ = 4.511 * |
|  | Pop(Found)*Gen | 3.0 | F_6,26_ = 0.303 n.s. |
|  | Body Size | 15.1 | F_1,26_ = 1.501 n.s. |
|  | Error | 10.0 |  |

Note: significance levels: *P*>0.1 n.s.; 0.1> *P* >0.05 m.s.; 0.05> *P* >0.01*; 0.01>*P* >0.001**; *P* <0.001 ***
