# Supplementary material for: Laboratory Selection Quickly Erases Historical Differentiation
Source: PLoS One. 2014 May 2;9(5):e96227. doi: 10.1371/journal.pone.0096227 (PMC4008540; doi:10.1371/journal.pone.0096227)
Supplement: Table S4 — Principal Component Analysis including all traits and generations. (DOCX) [file pone.0096227.s004.docx]

**Table S4.** Principal Component Analysis including all traits and generations

A) Eigenvectors for the first three axes of Principal Component Analysis, using all populations, traits and generations

| Trait | PC 1 | PC 2 | PC 3 |
| --- | --- | --- | --- |
| A1R | 0.544 | 0.092 | 0.056 |
| F1-7 | 0.554 | 0.235 | -0.002 |
| F8-12 | 0.547 | 0.178 | 0.142 |
| RF | 0.192 | -0.819 | 0.529 |
| BS | 0.249 | -0.483 | -0.835 |

Note: PC 1 explains 59.48 % of the total variation, PC2 and PC3 explain 19.50 % and 16.84 %, respectively.

B) Euclidean Distances calculated from Principal Components Analysis between all foundations at generation 6 and 22

| Gen | Regimes | Euclidean Distance | Lower and Upper 95% limits | |
| --- | --- | --- | --- | --- |
| 6 | Ad-Mo | 2.8464*** | 2.8737 | 5.2773 |
|  | Ad-Gro | 3.3964*** | 2.9162 | 4.1564 |
|  | Gro-Mo | 5.3533*** | 5.0671 | 5.9581 |
| 22 | Ad-Mo | 0.5953 n.s. | 0.25942 | 2.31401 |
|  | Ad-Gro | 0.8779 n.s. | 0.50877 | 2.51833 |
|  | Gro-Mo | 1.1097 n.s. | 0.64736 | 2.07378 |

Note: significance levels: *P*>0.1 n.s; *P*<0.001 ***
