# Supplementary material for: Laboratory Selection Quickly Erases Historical Differentiation
Source: PLoS One. 2014 May 2;9(5):e96227. doi: 10.1371/journal.pone.0096227 (PMC4008540; doi:10.1371/journal.pone.0096227)
Supplement: Table S5 — Variance components of History and Chance for Early Fecundity and Starvation Resistance. (DOCX) [file pone.0096227.s005.docx]

**Table S5**. Variance components of History and Chance for Early Fecundity (A) and Starvation Resistance (B).

A) Early Fecundity

| Gen | Model parameters | MS | F_(df1, df2)_ | σ^2^ | Direct Estimate | Bootstrap Estimate - average | Lower and Upper 95% limits | | √σ^2^/M | Direct Estimate | Bootstrap Estimate - average | Lower and Upper 95% limits | |
| --- | --- | --- | --- | --- | --- | --- | --- | --- | --- | --- | --- | --- | --- |
| 6 | Found | 57089.0 | F_2,6_ = 37.886 *** | σ^2^_History_ | 786.659 | 812.212 | 549.786 | 1069.653 | σ^2^_History_ | 0.645 | 0.653 | 0.572 | 0.724 |
|  | Pop(Found) | 1507.0 | F_6,203_ = 1.942 m.s. | σ^2^_Chance_ | 31.034 | 63.866 | -6.9725 | 177.243 | σ^2^_Chance_ | 0.128 | 0.185 | 0 | 0.308 |
|  | Error | 776.1 |  |  |  |  |  |  |  |  |  |  |  |
| 11 | Found | 12215.5 | F_2,6_ = 4.559 m.s. | σ^2^_History_ | 183.441 | 226.830 | 50.803 | 514.289 | σ^2^_History_ | 0.260 | 0.293 | 0.130 | 0.476 |
|  | Pop(Found) | 2680.8 | F_6,147_ = 2.980 ** | σ^2^_Chance_ | 102.792 | 158.845 | 36.503 | 324.432 | σ^2^_Chance_ | 0.194 | 0.240 | 0.113 | 0.343 |
|  | Error | 899.5 |  |  |  |  |  |  |  |  |  |  |  |
| 14 | Found | 2403.8 | F_2,6_ =1.136 n.s. | σ^2^_History_ | 5.419 | 42.302 | -17.659 | 230.161 | σ^2^_History_ | 0.036 | 0.104 | 0 | 0.263 |
|  | Pop(Found) | 2115.8 | F_6,150_ = 2.428 * | σ^2^_Chance_ | 70.452 | 121.173 | 7.206 | 277.293 | σ^2^_Chance_ | 0.130 | 0.171 | 0.041 | 0.263 |
|  | Error | 871.5 |  |  |  |  |  |  |  |  |  |  |  |
| 18 | Found | 1578.0 | F_2,6_ = 0.522 n.s. | σ^2^_History_ | -11.875 | 34.453 | -31.033 | 247.536 | σ^2^_History_ | 0 | 0.109 | 0 | 0.278 |
|  | Pop(Found) | 2180.4 | F_6,145_ = 2.268 * | σ^2^_Chance_ | 71.395 | 128.719 | 24.487 | 275.862 | σ^2^_Chance_ | 0.165 | 0.224 | 0.096 | 0.332 |
|  | Error | 961.3 |  |  |  |  |  |  |  |  |  |  |  |
| 22 | Found | 2344.4 | F_2,6_ =0.880 n.s. | σ^2^_History_ | -4.812 | 38.243 | -29.059 | 135.572 | σ^2^_History_ | 0 | 0.113 | 0 | 0.213 |
|  | Pop(Found) | 2666.8 | F_6,196_ = 2.974 ** | σ^2^_Chance_ | 77.808 | 115.109 | 17.679 | 240.864 | σ^2^_Chance_ | 0.157 | 0.193 | 0.072 | 0.282 |
|  | Error | 896.7 |  |  |  |  |  |  |  |  |  |  |  |

Note: significance levels: *P*>0.1 n.s.; 0.1>*P*>0.05 m.s.; 0.05>*P*>0.01*; 0.01>*P*>0.001**; *P*<0.001 ***

B) Starvation Resistance

| Gen | Model parameters | MS | F_(df1, df2)_ | σ^2^ | Direct Estimate | Bootstrap Estimate -average | Lower and Upper 95% limits | | √σ^2^/M | Direct Estimate | Bootstrap Estimate - average | Lower and Upper 95% limits | |
| --- | --- | --- | --- | --- | --- | --- | --- | --- | --- | --- | --- | --- | --- |
| 6 | Found | 978.5 | F_2,6_ = 6.664 * | σ^2^_History_ | 12.550 | 14.523 | 7.712 | 23.002 | σ^2^_History_ | 0.093 | 0.100 | 0.071 | 0.130 |
|  | Pop(Found) | 147.0 | F_6,190_ = 2.096 m.s. | σ^2^_Chance_ | 3.480 | 6.606 | 0.138 | 16.245 | σ^2^_Chance_ | 0.049 | 0.068 | 0.010 | 0.106 |
|  | Error | 70.1 |  |  |  |  |  |  |  |  |  |  |  |
| 11 | Found | 93.4 | F_2,6_ = 0.388 n.s. | σ^2^_History_ | -3.252 | 1.789 | -5.307 | 15.276 | σ^2^_History_ | 0 | 0.033 | 0 | 0.098 |
|  | Pop(Found) | 241.3 | F_6,128_ = 2.276 * | σ^2^_Chance_ | 8.900 | 15.621 | 0.997 | 37.471 | σ^2^_Chance_ | 0.076 | 0.101 | 0.025 | 0.158 |
|  | Error | 106.1 |  |  |  |  |  |  |  |  |  |  |  |
| 14 | Found | 3.6 | F_2,6_ = 0.023 n.s. | σ^2^_History_ | -2.940 | 0.026 | -3.184 | 9.572 | σ^2^_History_ | 0 | 0.005 | 0 | 0.080 |
|  | Pop(Found) | 154.4 | F_6,145_ = 3.349 ** | σ^2^_Chance_ | 6.329 | 8.834 | 1.314 | 20.172 | σ^2^_Chance_ | 0.062 | 0.073 | 0.028 | 0.112 |
|  | Error | 46.1 |  |  |  |  |  |  |  |  |  |  |  |
| 18 | Found | 61.1 | F_2,6_ = 0.187 n.s. | σ^2^_History_ | -5.399 | 0.829 | -6.747 | 16.834 | σ^2^_History_ | 0 | 0.022 | 0 | 0.104 |
|  | Pop(Found) | 327.8 | F_6,141_ = 4.587 *** | σ^2^_Chance_ | 15.443 | 20.190 | 7.072 | 38.118 | σ^2^_Chance_ | 0.101 | 0.116 | 0.067 | 0.161 |
|  | Error | 71.5 |  |  |  |  |  |  |  |  |  |  |  |
| 22 | Found | 39.8 | F_2,6_ = 0.184 n.s. | σ^2^_History_ | -2.625 | 0.659 | -2.784 | 10.531 | σ^2^_History_ | 0 | 0.001 | 0 | 0.094 |
|  | Pop(Found) | 216.4 | F_6,194_ = 3.981 *** | σ^2^_Chance_ | 7.201 | 9.552 | 1.707 | 20.965 | σ^2^_Chance_ | 0.073 | 0.085 | 0.035 | 0.127 |
|  | Error | 54.4 |  |  |  |  |  |  |  |  |  |  |  |

Note: significance levels: *P*>0.1 n.s.; 0.1>*P*>0.05 m.s.; 0.05>*P*>0.01*; 0.01>*P*>0.001**; *P*<0.001 ***

Estimates were obtained from ANOVA model Y = μ + Foundation + Population(Foundation) + ε. Non-standardized (σ^2^) and standardized (√σ^2^/M) values are shown. Estimates were standardized by the square of the mean values of all populations involved in the estimates (see Material and Methods and Appendix S1). Prior to standardization, negative values were changed to zero.
