# Supplementary material for: Laboratory Selection Quickly Erases Historical Differentiation
Source: PLoS One. 2014 May 2;9(5):e96227. doi: 10.1371/journal.pone.0096227 (PMC4008540; doi:10.1371/journal.pone.0096227)
Supplement: Table S6 — Variance components of Selection for Early Fecundity and Starvation Resistance. (DOCX) [file pone.0096227.s006.docx]

**Table S6.** Variance components of Selection for Early Fecundity (F1-7) and Starvation Resistance (RF) for Adraga (A), Montpellier (B) and Groningen (C)

A) Adraga

|  |  |  |  |  | σ^2^ | | | | √σ^2^/M | | | |
| --- | --- | --- | --- | --- | --- | --- | --- | --- | --- | --- | --- | --- |
| Trait | Gen | Model Parameters | MS | F_df1,df2_ | Direct Estimate | Bootstrap Estimate - average | Lower and Upper 95% limits | | Direct Estimate | Bootstrap Estimate - average | Lower and Upper 95% limits | |
| F1-7 | 6 - 11 | Pop | 480.136 | F_2,2_ = 0.108 n.s. |  |  |  |  |  |  |  |  |
|  |  | Gen | 1170.530 | F_1,2_ = 0.263 n.s. | -55.362 | 18.239 | -95.804 | 424.864 | 0 | 0.049 | 0 | 0.240 |
|  |  | Pop*Gen | 4447.562 | F_2,113_ = 3.943 * |  |  |  |  |  |  |  |  |
|  |  | Error | 1127.974 |  |  |  |  |  |  |  |  |  |
|  | 6 - 14 | Pop | 42.718 | F_2,2_ = 0.023 n.s. |  |  |  |  |  |  |  |  |
|  |  | Gen | 33514.300 | F_1,2_ = 18.176 m.s. | 521.006 | 548.102 | 278.299 | 1181.786 | 0.263 | 0.270 | 0.191 | 0.397 |
|  |  | Pop*Gen | 1843.962 | F_2,116_ = 1.720 n.s. |  |  |  |  |  |  |  |  |
|  |  | Error | 1072.206 |  |  |  |  |  |  |  |  |  |
|  | 6 - 18 | Pop | 4238.033 | F_2,2_ = 1.940 n.s. |  |  |  |  |  |  |  |  |
|  |  | Gen | 38344.096 | F_1,2_ = 17.566 m.s. | 605.692 | 644.200 | 271.469 | 1379.499 | 0.315 | 0.322 | 0.211 | 0.445 |
|  |  | Pop*Gen | 2184.301 | F_2,114_ = 2.061 n.s. |  |  |  |  |  |  |  |  |
|  |  | Error | 1059.985 |  |  |  |  |  |  |  |  |  |
|  | 6 - 22 | Pop | 4297.272 | F_2,2_ = 1.320 n.s. |  |  |  |  |  |  |  |  |
|  |  | Gen | 57219.345 | F_1,2_ = 17.586 m.s. | 788.052 | 834.847 | 289.681 | 1678.111 | 0.369 | 0.377 | 0.227 | 0.503 |
|  |  | Pop*Gen | 3254.487 | F_2,131_ = 2.635 m.s. |  |  |  |  |  |  |  |  |
|  |  | Error | 1235.033 |  |  |  |  |  |  |  |  |  |
| RF | 6 - 11 | Pop | 174.116 | F_2,2_ = 0.925 n.s. |  |  |  |  |  |  |  |  |
|  |  | Gen | 29.644 | F_1,2_ = 0.157 n.s. | -3.023 | 0.611 | -4.742 | 15.764 | 0 | 0.020 | 0 | 0.103 |
|  |  | Pop*Gen | 188.198 | F_2,100_ = 1.818 n.s. |  |  |  |  |  |  |  |  |
|  |  | Error | 103.505 |  |  |  |  |  |  |  |  |  |
|  | 6 - 14 | Pop | 302.651 | F_2,2_ = 3.377 n.s. |  |  |  |  |  |  |  |  |
|  |  | Gen | 29.828 | F_1,2_ = 0.333 n.s. | -1.016 | 0.388 | -1.327 | 4.101 | 0 | 0.017 | 0 | 0.055 |
|  |  | Pop*Gen | 89.622 | F_2,116_ = 1.155 n.s. |  |  |  |  |  |  |  |  |
|  |  | Error | 77.616 |  |  |  |  |  |  |  |  |  |
|  | 6 - 18 | Pop | 718.125 | F_2,2_ =5.831 n.s. |  |  |  |  |  |  |  |  |
|  |  | Gen | 173.060 | F_1,2_ = 1.407 n.s. | 0.872 | 2.849 | -2.223 | 14.047 | 0.024 | 0.045 | 0 | 0.104 |
|  |  | Pop*Gen | 123.165 | F_2,109_ = 1.558 n.s. |  |  |  |  |  |  |  |  |
|  |  | Error | 79.058 |  |  |  |  |  |  |  |  |  |
|  | 6 - 22 | Pop | 562.999 | F_2,2_ = 2.282 n.s. |  |  |  |  |  |  |  |  |
|  |  | Gen | 5.160 | F_1,2_ = 0.021 n.s. | -3.663 | 0.264 | -3.901 | 13.271 | 0 | 0.014 | 0 | 0.101 |
|  |  | Pop*Gen | 246.749 | F_2,126_ = 3.070 * |  |  |  |  |  |  |  |  |
|  |  | Error | 80.366 |  |  |  |  |  |  |  |  |  |

Note: significance levels: *P*>0.1 n.s.; 0.1>*P*>0.05 m.s.; 0.05>*P*>0.01*; 0.01>*P*>0.001**; *P*<0.001 ***

B) Montpellier

|  |  |  |  |  | σ^2^ | | | | √σ^2^/M | | | |
| --- | --- | --- | --- | --- | --- | --- | --- | --- | --- | --- | --- | --- |
| Trait | Gen | Model Parameters | MS | F_df1,df2_ | Direct Estimate | Bootstrap Estimate - average | Lower and Upper 95% limits | | Direct Estimate | Bootstrap Estimate - average | Lower and Upper 95% limits | |
| F1-7 | 6 - 11 | Pop | 2309.862 | F_2,2_ =1.258 n.s. |  |  |  |  |  |  |  |  |
|  |  | Gen | 21176.301 | F_1,2_ = 11.533 m.s. | 310.606 | 339.612 | 99.661 | 821.021 | 0.250 | 0.259 | 0.151 | 0.390 |
|  |  | Pop*Gen | 1836.250 | F_2,119_ = 3.658 * |  |  |  |  |  |  |  |  |
|  |  | Error | 501.960 |  |  |  |  |  |  |  |  |  |
|  | 6 - 14 | Pop | 366.745 | F_2,2_ = 1.779 n.s. |  |  |  |  |  |  |  |  |
|  |  | Gen | 103038.334 | F_1,2_ = 499.837 ** | 1680.265 | 1683.764 | 1494.586 | 1981.690 | 0.551 | 0.551 | 0.526 | 0.598 |
|  |  | Pop*Gen | 206.136 | F_2,117_ = 0.389 n.s. |  |  |  |  |  |  |  |  |
|  |  | Error | 530.185 |  |  |  |  |  |  |  |  |  |
|  | 6 - 18 | Pop | 1244.346 | F_2,2_ = 2.237 n.s. |  |  |  |  |  |  |  |  |
|  |  | Gen | 119955.303 | F_1,2_ = 215.612 ** | 1917.575 | 1928.576 | 1442.245 | 2237.092 | 0.661 | 0.662 | 0.598 | 0.714 |
|  |  | Pop*Gen | 556.348 | F_2,119_ = 1.087 n.s. |  |  |  |  |  |  |  |  |
|  |  | Error | 512.053 |  |  |  |  |  |  |  |  |  |
|  | 6 - 22 | Pop | 1080.987 | F_2,2_ = 9.214 m.s. |  |  |  |  |  |  |  |  |
|  |  | Gen | 189074.779 | F_1,2_ = 1609.906 *** | 2699.943 | 2703.389 | 2543.432 | 2976.934 | 0.792 | 0.792 | 0.782 | 0.798 |
|  |  | Pop*Gen | 117.325 | F_2,134_ = 0.266 n.s. |  |  |  |  |  |  |  |  |
|  |  | Error | 441.569 |  |  |  |  |  |  |  |  |  |
| RF | 6 - 11 | Pop | 361.917 | F_2,2_ = 2.992 n.s. |  |  |  |  |  |  |  |  |
|  |  | Gen | 196.319 | F_1,2_ = 1.623 n.s. | 1.339 | 3.290 | -2.098 | 15.983 | 0.031 | 0.047 | 0 | 0.103 |
|  |  | Pop*Gen | 120.955 | F_2,107_ = 1.236 n.s. |  |  |  |  |  |  |  |  |
|  |  | Error | 97.883 |  |  |  |  |  |  |  |  |  |
|  | 6 - 14 | Pop | 64.994 | F_2,2_ = 48.564 * |  |  |  |  |  |  |  |  |
|  |  | Gen | 320.337 | F_1,2_ = 233.833 ** | 5.612 | 5.613 | 4.558 | 7.161 | 0.063 | 0.063 | 0.055 | 0.071 |
|  |  | Pop*Gen | 1.338 | F_2,108_ = 0.021 n.s. |  |  |  |  |  |  |  |  |
|  |  | Error | 64.218 |  |  |  |  |  |  |  |  |  |
|  | 6 - 18 | Pop | 376.890 | F_2,2_ = 1.495 n.s. |  |  |  |  |  |  |  |  |
|  |  | Gen | 28.472 | F_1,2_ = 0.113 n.s. | -3.863 | 0.296 | -3.895 | 12.403 | 0 | 0.012 | 0 | 0.096 |
|  |  | Pop*Gen | 252.034 | F_2,110_ = 3.769 * |  |  |  |  |  |  |  |  |
|  |  | Error | 66.863 |  |  |  |  |  |  |  |  |  |
|  | 6 - 22 | Pop | 126.740 | F_2,2_ = 7.341 n.s. |  |  |  |  |  |  |  |  |
|  |  | Gen | 268.991 | F_1,2_ = 15.521 m.s. | 3.818 | 4.148 | 0.968 | 6.916 | 0.054 | 0.056 | 0.028 | 0.071 |
|  |  | Pop*Gen | 17.265 | F_2,126_ = 0.264 n.s. |  |  |  |  |  |  |  |  |
|  |  | Error | 65.407 |  |  |  |  |  |  |  |  |  |

Note: significance levels: *P*>0.1 n.s.; 0.1>*P*>0.05 m.s.; 0.05>*P*>0.01*; 0.01>*P*>0.001**; *P*<0.001 ***

C) Groningen

|  |  |  |  |  | σ^2^ | | | | √σ^2^/M | | | |
| --- | --- | --- | --- | --- | --- | --- | --- | --- | --- | --- | --- | --- |
| Trait | Gen | Model Parameters | MS | F_df1,df2_ | Direct Estimate | Bootstrap Estimate - average | Lower and Upper 95% limits | | Direct Estimate | Bootstrap Estimate - average | Lower and Upper 95% limits | |
| F1-7 | 6 - 11 | Pop | 3289.060 | F_2,2_ =5.015 n.s. |  |  |  |  |  |  |  |  |
|  |  | Gen | 29.673 | F_1,2_ = 0.045 n.s. | -10.154 | 0.714 | -10.499 | 33.593 | 0 | 0.009 | 0 | 0.062 |
|  |  | Pop*Gen | 655.886 | F_2,118_ = 0.754 n.s. |  |  |  |  |  |  |  |  |
|  |  | Error | 869.281 |  |  |  |  |  |  |  |  |  |
|  | 6 - 14 | Pop | 2371.854 | F_2,2_ = 0.377 n.s. |  |  |  |  |  |  |  |  |
|  |  | Gen | 434.874 | F_1,2_ = 0.069 n.s. | -93.370 | 7.953 | -94.183 | 282.636 | 0 | 0.030 | 0 | 0.196 |
|  |  | Pop*Gen | 6293.187 | F_2,120_ = 7.414 *** |  |  |  |  |  |  |  |  |
|  |  | Error | 848.784 |  |  |  |  |  |  |  |  |  |
|  | 6 - 18 | Pop | 1019.241 | F_2,2_ = 0.527 n.s. |  |  |  |  |  |  |  |  |
|  |  | Gen | 3364.416 | F_1,2_ = 1.739 n.s. | 23.800 | 57.661 | 0.243 | 348.999 | 0.060 | 0.095 | 0.006 | 0.238 |
|  |  | Pop*Gen | 1935.458 | F_2,115_ = 1.933 n.s. |  |  |  |  |  |  |  |  |
|  |  | Error | 1001.305 |  |  |  |  |  |  |  |  |  |
|  | 6 - 22 | Pop | 454.359 | F_2,2_ = 0.139 n.s. |  |  |  |  |  |  |  |  |
|  |  | Gen | 30647.314 | F_1,2_ = 9.406 m.s. | 391.431 | 443.652 | 176.389 | 1187.112 | 0.240 | 0.257 | 0.158 | 0.427 |
|  |  | Pop*Gen | 3259.465 | F_2,134_ = 3.888 * |  |  |  |  |  |  |  |  |
|  |  | Error | 838.308 |  |  |  |  |  |  |  |  |  |
| RF | 6 - 11 | Pop | 146.161 | F_2,2_ = 0.702 n.s. |  |  |  |  |  |  |  |  |
|  |  | Gen | 370.813 | F_1,2_ = 1.783 n.s. | 2.808 | 6.351 | -0.846 | 20.324 | 0.043 | 0.065 | 0 | 0.116 |
|  |  | Pop*Gen | 208.061 | F_2,111_ = 3.801 * |  |  |  |  |  |  |  |  |
|  |  | Error | 54.741 |  |  |  |  |  |  |  |  |  |
|  | 6 - 14 | Pop | 208.898 | F_2,2_ = 0.763 n.s. |  |  |  |  |  |  |  |  |
|  |  | Gen | 513.732 | F_1,2_ = 1.877 n.s. | 3.993 | 8.297 | 0.153 | 24.192 | 0.050 | 0.074 | 0.010 | 0.127 |
|  |  | Pop*Gen | 273.652 | F_2,115_ = 7.185 ** |  |  |  |  |  |  |  |  |
|  |  | Error | 38.087 |  |  |  |  |  |  |  |  |  |
|  | 6 - 18 | Pop | 4.528 | F_2,2_ = 26.070 * |  |  |  |  |  |  |  |  |
|  |  | Gen | 1977.699 | F_1,2_ = 6350.349 *** | 33.824 | 33.864 | 33.331 | 34.882 | 0.147 | 0.147 | 0.146 | 0.150 |
|  |  | Pop*Gen | 0.174 | F_2,110_ = 0.003 n.s. |  |  |  |  |  |  |  |  |
|  |  | Error | 66.325 |  |  |  |  |  |  |  |  |  |
|  | 6 - 22 | Pop | 48.420 | F_2,2_ = 0.549 n.s. |  |  |  |  |  |  |  |  |
|  |  | Gen | 735.930 | F_1,2_ = 8.347 n.s. | 9.394 | 10.697 | 4.448 | 18.810 | 0.081 | 0.087 | 0.055 | 0.115 |
|  |  | Pop*Gen | 88.228 | F_2,132_ = 2.116 n.s. |  |  |  |  |  |  |  |  |
|  |  | Error | 41.693 |  |  |  |  |  |  |  |  |  |

Note: significance levels: *P*>0.1 n.s.; 0.1>*P*>0.05 m.s.; 0.05>*P*>0.01*; 0.01>*P*>0.001**; *P*<0.001 ***

Non-standardized (σ^2^) and standardized (√σ^2^/M) values are shown. Estimates were standardized by the square of the mean values of all populations involved in the estimates (see Material and Methods and Supplementary Information). Prior to standardization, negative values were changed to zero.
