# Supplementary material for: Laboratory Selection Quickly Erases Historical Differentiation
Source: PLoS One. 2014 May 2;9(5):e96227. doi: 10.1371/journal.pone.0096227 (PMC4008540; doi:10.1371/journal.pone.0096227)
Supplement: Table S7 — Comparisons of the initial differences between the three foundations, relative to differences in previous studies. (DOCX) [file pone.0096227.s007.docx]

**Table S7.** Comparisons of the initial differences between the Adraga, Montpellier and Groningen foundations, relative to differences in previous studies between close-by or the same location.

| Year of Foundation | Comparison | Generation | σ^2^ | √σ^2^/M |
| --- | --- | --- | --- | --- |
| 2010 | Ad_Mo_Gro | 6 | 786.659 | 0.645 |
| 2001 | AR_TW | 4 | 42.160 | 0.152 |
| 2005 | FWA_FWB | 3 | 25.411 | 0.197 |
|  | FWA_NARA | 3 | 0 | 0 |
|  | FWA_NARB | 3 | 0 | 0 |
|  | FWB_NARA | 3 | 86.675 | 0.347 |
|  | FWB_NARB | 3 | 92.782 | 0.356 |
|  | NARA_NARB | 3 | 0 | 0 |

Variance components for the 2001 and 2005 data reflect the importance of sampling effects (Simões *et al*. 2008; Santos *et al*. 2012), while the present study (2010) reflects the impact of history associated with populations from contrasting latitudes in the European Continent.
