# Supplementary material for: Laboratory Selection Quickly Erases Historical Differentiation
Source: PLoS One. 2014 May 2;9(5):e96227. doi: 10.1371/journal.pone.0096227 (PMC4008540; doi:10.1371/journal.pone.0096227)
Supplement: Additional Methods S1 — (DOCX) [file pone.0096227.s008.docx]

Additional Methods

*Preliminary assay*

In the 4^th^ generation a preliminary assay on adult life history traits was done with all experimental foundations (not replicated) and controls, both developed in normal medium and medium with ceftriaxone/ spectinomycin, which gave no significant interactions between treatments and foundations.

*Estimation of Body Size*

As a proxy for body size, we measured wing size. For the wing size measurements, x and y coordinates of 13 morphological landmarks of the wing were recorded by using the Fly Wing 15Lmk plug-in implemented in IMAGEJ 1.33u software (<http://rsb.info.nih.gov/ij/>). Wing size was estimated as centroid size, defined as the square root of the sum of the 26 squared Euclidian distances of the 13 landmarks to the centroid – see [1]. Analyses were performed using the log-transformed centroid size values.

*Estimates of causal components of variation*

To address the relative role of History, Chance and Selection we estimated the variance components associated with these different sources of variation for early fecundity and starvation resistance. As a note, for simplification we will refer as “variance component” to the estimates of the causal contributions to variation of a given ANOVA factor for both fixed and random factors since estimates are equivalent. Using the approximation described by [2], we used the Mean Squares (MS) of the nested ANOVA model of each generation (described in Methods) to estimate the variance components of history – variation due to differences among foundations (σ^2^_History_) - and chance – variation due to differences among populations within foundations (σ^2^_Chance_) - as:

$\sigma^{2}History=\frac{MSFound-MSError-(n'0\sigma^{2}Chance)}{nb0}$,

,

Where *n*_0_, *n*'_0_ and *nb*_0_ are the corrections applied to a nested ANOVA with unequal sample sizes as defined in [2] (pg. 297).

To calculate the cumulative effect of selection - variation between the earliest generation assayed and each one of the later generations (σ^2^_Selection_) - for each Foundation and pair of generations we applied mixed bifactorial ANOVAS with the following model:

$$Y=\mu+Gen+Pop+Gen*Pop+\varepsilon,$$

where *Y* refers to Early Fecundity or Starvation Resistance, *Gen* refers to the two generations of a given comparison (generation 6 *versus* generation 11, 14, 18 or 22), and *Pop* (random factor) to the three replicate populations of that Foundation. In this analysis we used as data points in each generation the differences between experimental populations and average values of controls. The MS of the ANOVA was then used to calculate the variance component of Selection as follows:

$\sigma^{2}Selection=\frac{MSGen-MSGen*Pop}{n'*b}$,

With *b* being the number of levels of the *Population* (*b*=3) and *n´* the corrected *n* (number of individual observations) for unequal sample sizes as used in [3], following [4]. The variance components estimates were also standardized by the square of the mean values of all populations involved in the estimates. In the case of variation due to selection, where the estimates involved differences to controls, values of both experimental and control populations were used in the estimation of the mean. For the sake of graphical presentation, estimates were also square root transformed. It is relevant to mention that Restricted Maximum Likelihood (REML) estimates, one of the preferred methods for estimating variance components, especially in unbalanced designs, provided similar results to those presented in this study.

In order to obtain confidence intervals for each of the variance components estimated, a bootstrap analysis was done (n=999) at the level of individuals within each replicate population for the variance component of chance, at the replicate population level within each foundation for the history variance component and at the replicate population level within each foundation and across generations for the selection variance component. All data analyses were performed using STATISTICA 10, EXCEL and R [5] with the car package [6].

**Literature Cited:**

1. Santos M, Iriarte PF, Céspedes W (2005) Genetics and geometry of canalization and developmental stability in *Drosophila subobscura*. BMC Evol Biol 5: 7.

2. Sokal RR, Rohlf FJ (1995) Biometry. 3rd ed. New York: W.H. Freeman and Co.

3. Joshi A, Castillo RB, Mueller LD (2003) The contribution of ancestry, chance, and past and ongoing selection to adaptive evolution. J Genet 82: 147–162.

4. Neter J, Wasserman W, Kutner MH (1990) Applied linear statistical models: regression, analysis of variance, and experimental design. 3nd ed. Boston: Irwin.

5. R CDT (2008) R: a language and environment for statistical computing. Version 2.70. R Found Stat Comput.

6. Fox J, Weisberg S (2011) An {R} Companion to Applied Regression. Thousand Oaks, CA: Sage.
